# Effects of IMOD^™^ on angiogenesis, *miR-503* and *CDC25* expression levels in heart tissue of diabetic male rats

**Published:** 2018

**Authors:** Arshad Ghaffari-Nasab, Fariba Mirzaie Bavil, Rafigheh Ghiasi, Saeed Sadigh-Eteghad, Mohammad Reza Alipour

**Affiliations:** 1 *Neurosciences Research Center, Tabriz University of Medical Sciences, Tabriz, Iran*; 2 *Drug Applied Research Center, Tabriz University of Medical Sciences, Tabriz, Iran*

**Keywords:** Diabetes, Angiogenesis, MiR-503, CDC25, IMOD

## Abstract

**Objective:**

Diabetes is associated with vascular complications and impaired angiogenesis. Since angiogenesis plays a crucial role in vascular homeostasis in ischemic heart diseases, in this study, the effect of IMOD™ on *miR-503* and *CDC25* expression level which are altered in impaired angiogenesis were investigated in heart tissue of diabetic rats.

**Materials and Methods:**

Forty male Wistar rats (200-250 g) were randomly classified into 4 groups: control (C), IMOD™ (I), diabetes (D), and diabetes+IMOD™ (D+I). For induction of experimental diabetes in animals, a single dose of streptozotocin (STZ; 60mg/kg) was injected intraperitoneally. After 8 weeks of treatment with IMOD™ (20 mg/kg/day), heart tissue samples were removed and used for measurement of *miR-503* and *CDC25* expression level as well as histological studies.

**Results:**

Results of this study showed that diabetes decreased heart tissue angiogenesis which was associated with increased *miR-503* and reduced *CDC25* expression levels (p<0.05) and IMOD™ could reduce the expression of *miR-503* and increase the expression of *CDC25* (p<0.05). Moreover, IMOD™ extensively induced angiogenesis in the heart tissue of diabetic group. However, IMOD™ had no significant effect on expressions of *miR-503* and CDC25, or angiogenesis in healthy rats.

**Conclusion:**

This study showed that IMOD™ is able to increase angiogenesis in the heart tissue of diabetic rats. The angiogenic effect of IMOD™ is associated with reduction of *miR-503 *expression and increased expression of *CDC25*.

## Introduction

Diabetes mellitus is a chronic metabolic disease diagnosed with an inability to produce insulin or presence of high resistance towards its function (Deepthi et al., 2017 ▶). Diabetes is associated with many medical complications as well as high economic and social costs (Alva et al., 2015 ▶). One of the most important macro-vascular complications of diabetes is the impairment of cardiac function which can be induced by chronic hyperglycemic conditions (Huynh et al., 2014 ▶). According to the reports, cardiovascular complications are the main causes of death in diabetic subjects (Forbes and Cooper, 2013 ▶). Angiogenesis, defined as generation of new vessels from pre-existing vessel beds, is an important process particularly during  myocardial ischemia (Carmeliet, 2005 ▶). It has been revealed that diabetes leads to disruption of angiogenesis (Xu et al., 2012 ▶).

The mechanisms through which diabetes disrupts angiogenesis are very complicated. In this case, few molecular mechanisms such as oxidative stress, endothelial dysfunction and changes in micro ribonucleic acids (miRNA) levels have been proposed (Leeper and Cooke, 2011; Liu et al., 2012). Also, diabetes can affect the angiogenesis by disrupting the synthesis of endothelium-derived nitric oxide (NO) which is required for angiogenic responses (Kolluru et al., 2012 ▶). It is known that diabetes is associated with decreased serum levels of NO as well as diminished myocardial capillary density (Khazaei and Salehi, 2013 ▶). Moreover, it has been shown that hyperglycemic conditions impair neovascularization in the ischemic heart tissues (Kota et al., 2012 ▶).

The miRNAs are a class of RNAs which play important regulatory roles in various cellular processes by regulation of the protein-encoding genes expression (Kong et al., 2009 ▶). These highly protected molecules play an essential role in many pathological conditions, such as vascular inflammation, smooth muscle plasticity, atherosclerosis, stem cell differentiation and endothelial cells apoptosis (Rippe et al., 2012 ▶). Previous studies have shown that irregularities in function of miRNAs can cause several diseases in humans (Chang and Mendell, 2007 ▶). Caporali et al. have reported that the expression of *miR-503* is increased in plasma and ischemic muscles of diabetic patients. They notified that *miR-503* is involved in angiogenesis impairment during ischemic condition in endothelial cells and also showed that over-expression of *miR-503* inhibits proliferation, migration, and network-formation of endothelial cells. Therefore, *miR-503* might be considered an inhibitor of post-ischemic new vessel formation in hyperglycemia, and it can be a potential therapeutic target for endothelial dysfunction in diabetes mellitus (Caporali et al., 2011 ▶). In eukaryotic cells, the cell cycle progression is carried out by cyclin-dependent kinases (CDKs) activity (Timofeev et al., 2012 ▶). In this manner, CDC25 is a phosphatase that drives cell cycle progression by dephosphorylating CDKs. It was known that *miR-503* is significantly increased in diabetic muscles which is inversely associated with *CDC25* expression. Furthermore, plasma levels of *miR-503* increase in patients with diabetes (Caporali et al., 2011 ▶). It is thus conceivable that *CDC25* gene is a target of *miR-503* that plays an essential role in reduction of growth factors in endothelial cells (Caporali et al., 2011 ▶). It has been further revealed that *miR-503* decreases the expression of *CDC25* through proteasome degradation and transcriptional inhibition (Sarkar et al., 2010). Caporali et al. also reported that over-expression of *miR-503* inhibits the migration and proliferation of endothelial cells by affecting CDC25 levels (Caporali et al., 2011 ▶).

Nowadays, many drugs are used for the treatment of diabetes mellitus, but the side effects of these drugs should be taken into account when taken for a long time. Additionally, the high cost of these drugs is another problem for patients who must use them for long periods. In recent years, a large numbers of studies have examined the importance of natural/herbal products in the treatment of diabetes mellitus. Most of the previous studies have reported the anti-diabetic effects of some of the herbal drugs compared to the synthetic drugs (Modak et al., 2007; Tabatabaei-Malazy et al., 2013). IMOD™, the commercial name of setarud, is a natural modulator of immune system, which is composed of selenium-enriched extracts of *Rosa canina*, *Utricia diocia *and *Tanacetum vulgare *(Paydary et al., 2012 ▶). It has been shown that IMOD™ has anti-inflammatory and immune system stimulatory properties, as well as protective effect against oxidative stress (Mohseni-Salehi-Monfared et al., 2010 ▶). *In vivo* and *in vitro* studies in animal models and human have shown that this drug reduces the levels of pro-inflammatory cytokines including TNF-α, IL-2, and INF-γ. Furthermore, the beneficial effects of IMOD™ have been confirmed in clinical settings for treatment of type I diabetes (Farhoudi et al., 2013 ▶). 

Regarding disrupted angiogenesis in diabetes mellitus and useful effect of IMOD™ observed in some studies, in this study, the effects of IMOD™ on angiogenesis, as well as *miR-503* and CDC25 expression levels which are involved in the regulation of angiogenesis, were investigated in a diabetes model.

## Materials and Methods

**Laboratory animals**

In this study, forty Wistar male rats (200-250 g) were purchased from Animal Conservation Center of Tabriz University of Medical Sciences, Tabriz, Iran. The animals were transferred to the laboratory 24 hr before the start of the study in order to adapt to the environment. Animals were kept at 22±2 °C with 12 hr/12 hr light/dark cycles. The rats were randomly divided into four groups of control (C), IMOD™ (I), diabetes (D) and diabetes+IMOD™ (D+I), and investigated for 8 weeks. The weight and blood glucose levels of all animals were measured at the beginning and at the end of the experiment. 

**Diabetes induction and IMOD™ administration**

For induction of the diabetes, a single intraperitoneal injection of 60 mg/kg streptozotocin (STZ) was used and 72 hr after the injection, type I diabetes was induced in the rats (Akbarzadeh et al., 2013). In order to confirm the induction of diabetes, a drop of blood was collected from animals tail and located on the glucometer tape (Boehringer Mannheim Indianapolis, IN);animals with blood glucose levels above 300 mg/dl were considered diabetic (Furman, 2015). IMOD™ (RosePharmed Co., Iran) was injected intraperitoneally (20 mg/kg/day) for 8 weeks (Mohseni-Salehi-Monfared et al., 2010 ▶; Mohammadirad et al., 2011 ▶).

**Sampling**

After induction of anesthesia using ketamine (40mg/kg) and xylazine (5mg/kg), the heart tissue samples were removed under standard conditions. The apex of the heart tissues were fixed in 10% formalin solution for histological studies. The rest of the heart tissues were immediately frozen in liquid nitrogen and kept for gene expression studies in a freezer at -70 ° C.

**RNA isolation, cDNA synthesis and** **real-time quantitative PCR**

Total RNA including mRNA and miRNA were extracted from the hearts using RNX-Plus solution kit (Fermentase, Cinagen Co. Iran) and miR-amp kit (Parsgenome Co. Iran), respectively according to the manufacturers’ instructions. NanoDrop 1000 (Thermo Scientific, Waltham, and Mass) was used for assessment of RNA quantity and purity. The *miR-503* and *CDC25 *expression levels were quantitatively assessed by real-time PCR. Primers’ sequences for each gene were shown in Table 1.

The real-time PCR was done using Rotor-Gene 3000 (Corbett Research, Australia). All experiments were performed in duplicates. Real-time quantification was done by measuring the fluorescence intensity following binding of the SYBR Green dye to double-stranded DNA at the end of each amplification cycle. The PCR thermal cycle conditions included an initial denaturation at 94 C for 5 min, followed by 40 cycles at 94 C for 20 sec, 60 C for 20 sec and 72 C for 30 sec. The relative amount of mRNA and miRNA for each target gene was calculated based on its threshold cycle (Ct) compared to the Ct of the housekeeping (reference) gene (*β-actin* and *miR-191*). The relative quantification was performed by 2^-∆∆^ Ct method(Alipour et al., 2013 ▶).

**Histological studies**

*Immunostaining for PECAM-1/ CD31*

For investigation of angiogenesis, heart tissues were fixed in 10% formalin immediately after excision. Then, 3-μm thick sections were cut and samples floated on charged glass slides. Tissue samples were deparaffinized in xylene and subsequently dehydrated using a graded series of ethanol. Slides were incubated sequentially in proteinase K and treated with 0.3% hydrogen peroxide for blocking endogenous peroxidase activity. Samples were incubated with the primary antibody CD31 (Santa Cruz, USA), as a marker of angiogenesis at 4°C, overnight. Then, sections were washed and incubated with standard avidin–biotin complex (Santa Cruz, USA) according to the manufacturer's instructions. Next, samples were incubated with DAB (di-amino-benzidine), as a chromagen and counterstained with Mayer's hematoxylin. Finally, sections were cleared in xylene, mounted in entellan and assessed by light microscope (Olympus BX 40, Japan). For assessment of immunostaining, the intensity of the staining was scored as 0 (<10%), 1 (10-25%), 2 (25-50%), 3 (50 -75%) and 4 (75-100%) (Mirzaei Bavil et al., 2015 ▶).

**Statistical analysis**

For statistical analysis, the SPSS16 software was used. To assess normal distribution, the Kolmogorov-Smirnov test was used and in order to compare the means between the studied groups, in case the data distribution was normal, the one-way ANOVA and Tukey's *post-hoc *Test were used. The Paired-samples T test was also used to assess differences in weight means before and after the experiment in each group. Differences between groups were considered significant if p<0.05. All data were expressed as mean ± SEM.

## Results

**Fasting blood sugar and body weight before and after** **IMOD™ administration**

Table 2 shows the effects of STZ-induced diabetes and 8-week IMOD treatment on mean body weight and fasting blood sugar (FBS). As shown in Table 2, at the end of the treatment, the body weight of diabetic groups reduced significantly (p<0.05) compared to control group. Our data showed that body weight was not affected by IMOD treatment in both diabetic and non-diabetic animals.

The level of FBS remained mainly unchanged in control and IMOD™ groups. There was a significant (p<0.01) elevation in FBS in diabetic groups after 72 hr compared to control group. However, administration of IMOD™ could not change FBS levels in IMOD™-treated healthy group; interestingly, it caused significant (p<0.05) reduction in FBS levels in IMOD™-treated diabetic rats compared to diabetic control (Table 2).

**Table1:** Primers’ sequences for each gene used in this study.

**Gene name**	**Accession number**	**Target sequence **
***CDC25***	NM_133571.1	F: AAGGCAAACCTGTTAAGTGTG ^a^ R: GGGTACACTTCAACATTCCAG
***rno-miR-503-5p***	MIMAT0003213	UAGCAGCGGGAACAGUACUGCAG ^b^

a Sequences were derived from NCBI (www.ncbi.nlm.nih.gov).

b Sequences were derived from miRBase (www.mirbase.org).

**Table 2:** Effect of diabetes and 8-week IMOD™ treatment on mean body weight (g) and FBS (mg/dl).

**Groups**	**Body weight (g)**		**FBS (mg/dl)**	
	**Before treatment**	**After treatment**	**Before treatment**	**After treatment**
**Control**	267.75 ± 5.88	268.37 ± 8.31	95.12 ± 2.68	98.87 ± 6.77
**IMOD™**	270.5 ± 7.50	272.88 ± 7.52	95.37 ± 2.22	91.87 ± 3.21
**Diabetes**	284.83 ± 6.74	192.17 ± 11.13 #	447.83 ± 28.98 #	515.50 ± 39.38 #
**Diabetes + IMOD™**	272.12 ± 6.49	197 ± 8.18 #	498.88 ± 18.37 #	379.1 ± 22.66 * #

Data are reported as mean ± SEM for each group (n=8).

# p<0.01 vs control group;

* p<0.01 vs diabetic group.

**Effects of diabetes and IMOD™ on** ***miR-503*** **expression in heart tissue**

**Figure1:**
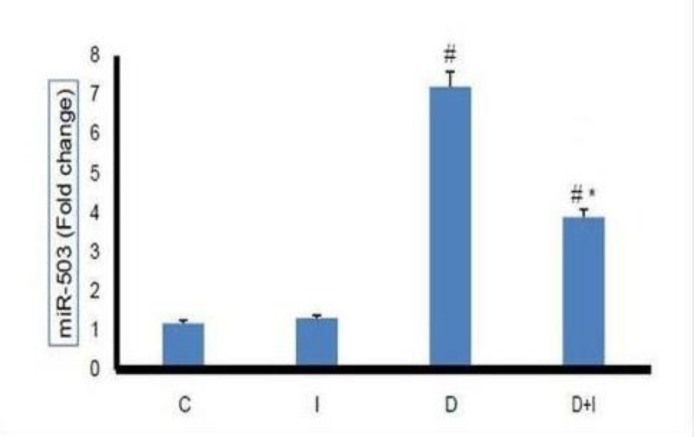
Effect of diabetes and 8-week IMOD™ treatment on *miR-503* expression in the heart tissue of experimental groups (n=8). (C): Control, (I): IMOD™ (20mg/kg/day), (D): Diabetes, (D+I): Diabetes+IMOD™. Data are normalized against *miR-191* and are shown as mean ± SEM.

In this study, it was shown that diabetes significantly increased *miR-503* expression in the heart tissue compared to control group (p<0.01). IMOD™ treatment (20 mg/kg/day for 8 weeks) was able to significantly reduce *miR-503* in diabetic animals (p<0.05). However, IMOD™ had no significant effect on *miR-503* expression in non-diabetic rats (Figure 1). 

#p<0.01 vs control group; * p<0.01 vs diabetic group.

**Effects of diabetes and IMOD**™** on *CDC25 *expression in heart tissue **

**Figure 2:**
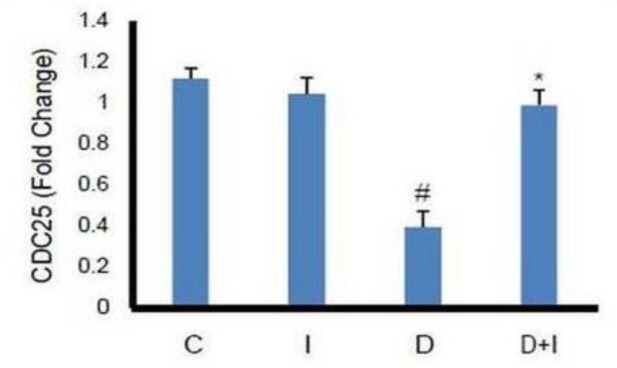
Effect of diabetes and 8-week IMOD™ treatment on *CDC25* gene expression in heart tissue of experimental groups (n=8). (C): Control, (I): IMOD™ (20mg/kg/day), (D): Diabetes, (D+I): Diabetes+IMOD™.

In diabetic rats, *CDC25* gene expression reduced significantly (p<0.01) as compared to control group and IMOD™ caused significant increases (p<0.01) in *CDC25* gene expression. IMOD™ had no significant impact in non-diabetic animals (Figure 2).

Data are normalized against *β-actin a*nd are shown as mean ± SEM. #p<0.01 vs control group; *p<0.01 vs diabetic group.

**Effects of diabetes and IMOD™ on angiogenesis in heart tissue**

**Figure 3:**
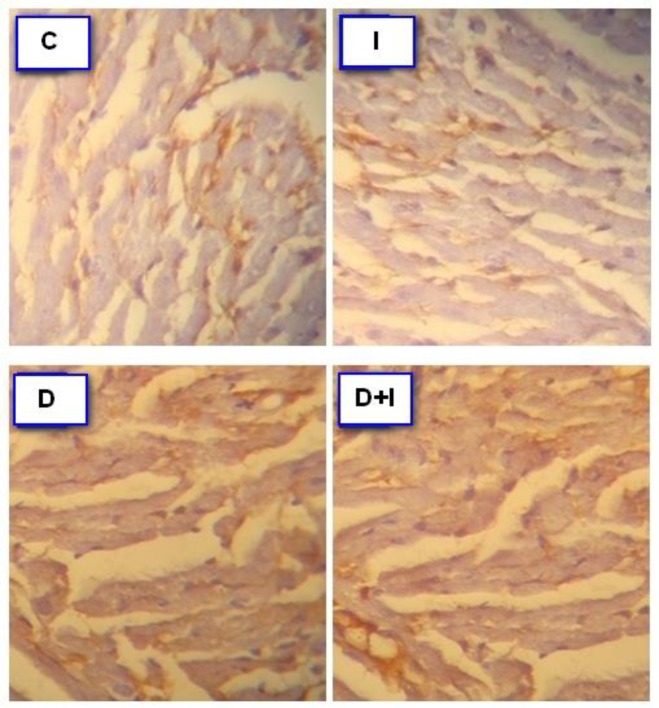
Immuno-histochemical detection of CD31 in heart tissue. Brown-stained tissues show CD31 immunostained endothelial cells in (C): Control, (I): IMOD™ (20mg/kg/day), (D): Diabetes, and (D+I): Diabetes+IMOD™. Treatment with IMOD™ increased angiogenesis compared to diabetes group (X40).

Immunostaining with CD31 marker was done for assessment of angiogenesis in heart tissue. Brown-stained tissues show CD31 immunostained endothelial cells. IMOD™ treatment had no effect on angiogenesis in non-diabetic animals, whereas it increased angiogenesis in heart tissue in diabetic rats (Figure 3).

## Discussion

This study was carried out to evaluate the effect of IMOD™ on levels of *miR-503* and *CDC25* expression and angiogenesis in heart tissue of STZ-induced diabetic rats. 

Data of this study showed that diabetes increased *miR-503* expression while reduced *CDC25* gene expression and angiogenesis in rats with type 1diabetes and administration of IMOD™ tended to restore these values to normal levels in diabetic animals. 

According to our data, diabetes impaired angiogenesis in heart tissue of STZ-treated rats. In agreement with the results of this study, another report showed that diabetes impairs angiogenesis and vascular homeostasis (Tahergorabi and Khazaei, 2012 ▶). Moreover, according to Abaci et al. findings, neovascularization in ischemic heart tissue is much lower in diabetic patients compared to healthy subjects (Abacı et al., 1999 ▶). Also, Leeper et al. showed that diabetes diminishes angiogenesis by disrupting the synthesis of NO and reducing vascular endothelial growth factor (*VEGF*) gene expression (Leeper and Cooke, 2011 ▶). In addition, another study indicated that diabetes can affect neovascularization in ischemic heart via reducing *VEGF* gene expression (Heather and Clarke, 2011). It is well known that VEGF is one of the factors that are involved in angiogenesis (Shibuya, 2011 ▶).

Interestingly, in this study, we found that IMOD™ improved angiogenesis in the heart tissue of diabetic rats. However, the results of the present study showed that IMOD™ had no significant effect on angiogenesis in normal animals. In this regard, Hormozi et al. reported that angiogenesis is increased by IMOD™ treatment in human ovarian tissue that transplanted into a nude mice model. They notified that IMOD™ may increase angiogenesis through increasing angiopoietin-1/angiopoietin-2 (Ang1/Ang2) ratio and VEGF level (Hormozi et al., 2015 ▶). Angiopoietin-1 (Ang1) has been reported to be necessary for vascular homeostasis through promoting maturation and integrity of vascular system after ischemia (Ikeoka et al., 2014 ▶). 

To find the mechanism through which IMOD™ promotes angiogenesis, we evaluated the expression levels of *miR-503* and its target gene, *CDC25*, in diabetic heart tissue. The results of the current experiment showed that IMOD™ decreased *miR-503* expression in diabetic animals. In agreement with our data, Caporali et al. revealed that hyperglycemia leads to increases in *miR-503* expression in the ischemic muscles of diabetic rats, so that, inhibition of *miR-503* function can effectively improve proliferation and migration of endothelial cells in diabetes. Their results also pointed out that the expression level of *CDC25* as a target of *miR-503*, is reduced in ischemic muscles of mice with STZ-induced diabetes (Caporali et al., 2011 ▶). As previously mentioned, CDC25 activates cell cycle progression through CDKs (Donzelli and Draetta, 2003 ▶) and up-regulation of *miR-503* inhibits the proliferation of endothelial cells by degradation and inhibition of CDC25 protein (Yamakuchi, 2012 ▶). In agreement with these studies, current study showed that hyperglycemia during diabetes increases *miR-503* expression, but reduces *CDC25* expression. Moreover, in this study, IMOD™ diminished *miR-503 *expression, while enhanced *CDC25* expression in diabetic rats. Therefore, it is likely that at least a part of angiogenic effect of IMOD™ is mediated through inhibition of *miR-503* expression and enhancement of *CDC25* expression. Further studies are needed to determine the effect of IMOD™ on angiogenesis concerning other factors such as NO in diabetes. Moreover, while inflammatory factors are involved in impairment of angiogenesis in hyperglycemia, it is conceivable that IMOD™ can also promote angiogenesis under diabetic conditions via its anti-inflammatory effects as already mentioned. Finally, It seems that the angiogenic effect of IMOD™ is also related to its selenium content which is reported as an angiogenic factor in several experiments (Bajpai et al., 2011 ▶).

It is concluded that IMOD™ can reduce *miR-503* expression while it can increase the expression of *CDC25* and augment angiogenesis in diabetic heart tissue. Thus, this herbal drug can be considered for alleviation of the vascular complications of diabetes, although more experiments are necessary to study other aspects of IMOD™ effect on diabetic complications.
